# Stress-induced changes in endogenous *TP53* mRNA 5′ regulatory region

**DOI:** 10.1016/j.jbc.2025.108418

**Published:** 2025-03-18

**Authors:** Jin Yeong Kim, Alexandra Furney, Brittany Benner, Arnab Sengupta

**Affiliations:** Department of Biological and Environmental Sciences, Georgia College & State University, Milledgeville, Georgia, USA

**Keywords:** mRNA, 5'UTR, cap-independent translation, SHAPE, cellular stress, RNA folding, IRES, ribosome

## Abstract

Tumor suppressor protein p53 is regulated in a number of ways, including during initiation of *TP53* mRNA translation. The 5′ end of *TP53* mRNA contains regulatory structures that enable noncanonical initiation using mechanisms that remain poorly described. Here we analyze per-nucleotide reactivity changes in the 5′ end secondary structure of *TP53* mRNA under in-cell conditions using A549 human lung carcinoma cells. We first construct a cell-free secondary structure model using SHAPE reagent 5-nitroisatoic anhydride on gently extracted and deproteinated RNA. We observe previously described regulatory features of the *TP53* mRNA 5′ end including two motifs which we refer to as long stem-loop (LSL) and short stem-loop (SSL), respectively. We observe a domain-forming helix that groups LSL and SSL, forming a three-helix junction. Applying in-cell selective 2′ hydroxyl acylation analyzed by primer extension and mutational profiling, we assess reactivity profiles with unstressed cells and with chemically induced stress conditions expected to stimulate *TP53* cap-independent translation. We analyze the effects of etoposide-induced DNA damage, CoCl_2_-induced hypoxia, and 5′ cap inhibition with 4EGI-1 treatment. Identifying stress-associated changes in the *TP53* 5′ end may help elucidate the role of regulatory RNA structure in cap-independent translation. Using ΔSHAPE, we identify in-cell protection sites that correspond with previously described RNA–protein binding sites on the apical loops of LSL and SSL. Furthermore, we identify several other potential interaction sites, some associated with specific types of stress. Some noteworthy changes include ΔSHAPE sites proximal to the start codons, at the three-helix junction and on the domain-forming helix. We summarize potential interactions on the cell-free secondary structure model.

Tumor protein 53 (p53) encoded in humans by the gene *TP53* is a widely studied transcription factor critical for response to cellular stress (1, 2). p53 promotes DNA damage repair, regulates cell cycle, inhibits cell proliferation, and induces apoptosis or senescence to prevent tumorigenesis (3, 4). In normal cells, p53 is maintained at low levels through regulators such as HDM2, and protein levels are elevated in response to certain types of stress (5, 6). Levels of cellular p53 are also regulated *via* translational control (7, 8, 9, 10). The 5′ end of *TP53* plays a crucial role in controlling translation initiation in a number of ways due to alternate promoter usage, alternate start codons, splicing isoforms, and regulatory RNA structures (2, 7, 10, 11, 12).

Under stress conditions like DNA damage, *TP53* undergoes noncanonical translation initiation (6, 13, 14). Previous studies have investigated the role of 5′ end secondary structure motifs and RNA-binding transacting factors in cap-independent p53 translation under cellular stress (15, 16, 17). From prior chemical probing and modeling studies, the structural context of the 5′ end regulatory region is established (10, 15, 16, 17). Using reporter assays, western blots, and mutational analysis, two putative internal ribosome entry sites (IRESs) have been identified (10, 12, 16, 18). The first is a long stem-loop (LSL) at positions 63 to 176 which contains the AUG1 start codon for full-length FLp53. This motif has been reported to bind polypyrimidine tract-binding protein (PTB) *in vitro* (16, 18, 19). The short stem-loop (SSL) at positions 187 to 225 reportedly facilitates translation using an alternate in-frame AUG start codon (position 260–2) encoding ΔN40p53, which lack 40 N-terminal amino acids that form transactivation domain 1 (11). Multiple RNA-binding proteins including PTB, annexin A2, death-associated protein 5, and HDM2/HDMX have been reported to interact with SSL, regulating translation of the ΔN40p53 isoform (16, 18, 20, 21). SSL has been previously described as a riboswitch-like structure (1).

Our understanding of the effect of cellular conditions on the mRNA structure of the endogenous *TP53* 5′ regulatory region and its interactions is limited. Here, we investigate the secondary structure of *TP53* 5′ end in A549 human lung carcinoma cells, a cell line used in prior studies to demonstrate increased p53 levels upon etoposide-induced DNA damage (22). We induce genotoxic stress by treating cells with etoposide, a topoisomerase-II inhibitor that specifically prevents DNA ligation activity leading to an accumulation of double-stranded breaks (23). DNA damage signals p53 activation in multiple ways, including cap-independent translational upregulation *via* interactions with binding proteins, such as MDM2 (mouse ortholog of HDM2) (6, 12, 24). We also evaluate the effect of hypoxic stress, which is associated with elevated p53 levels (25, 26, 27, 28). Hypoxic conditions were mimicked by treating cells with CoCl_2_, which binds to and blocks the oxygen-dependent degradation domain of HIF1a and has been shown to lead to p53 accumulation (25, 28, 29). Hypoxia-associated elevation of p53 levels is mainly due to inhibited degradation of p53 (25). Additionally, HIF1a also stimulates MDM2, which is known to upregulate *TP53* mRNA translation reportedly *via* 5′ UTR binding proteins (28, 30). Under genotoxic and hypoxic stress, *TP53* is likely to be translated using noncanonical initiation mechanisms (1). We also investigate *TP53* mRNA from cells treated with cap-inhibitor 4EGI-1, which blocks the interaction between eIF4E and eIF4G, a critical prerequisite for canonical cap-dependent translation initiation (31). Conditions favoring cap-independent translation initiation in *TP53* potentially involve changes in 5′ end structure and its interactions with transacting factors. Identifying 5′ end changes common to all in-cell conditions as well as condition-specific changes may help better describe the role of regulatory RNA structures in cap-independent translation of *TP53*.

We apply the SHAPE-MaP (selective 2′ hydroxyl acylation analyzed by primer extension and mutational profiling) chemical probing strategy to investigate the *TP53* mRNA 5′ end using the SHAPE reagent, 5-nitroisatoic anhydride (5NIA) (32, 33, 34, 35). Using an RT-mutate strategy, locations of flexible nucleotides on the RNA target region are recorded as mutations and analyzed alongside an untreated reference using massively paralleled sequencing (36). First, we use a rigorously validated strategy to obtain gently deproteinated, extracted RNA to conduct SHAPE-MaP under buffered conditions that favor native RNA folding (33, 37). The cell-free SHAPE reactivity data are used to build a secondary structure model for visualizing all subsequent comparative analysis (Fig. 1). Next, we probe live cells directly with the membrane-penetrable 5NIA SHAPE reagent generating per-nucleotide reactivity profiles with and without chemically induced stress. It is important to note that in-cell SHAPE-MaP data are affected by cellular interactions. Changes observed in base-pairing probabilities need to be interpreted cautiously as these are a likely consequence of masked reactivity at sites with cellular interactions. We apply ΔSHAPE analysis to compare per-nucleotide reactivity changes relative to the cell-free condition as well as relative to the in-cell unstressed condition. This strategy enables identification of ΔSHAPE protection and enhancement sites likely formed as a result of cellular interactions such as RNA–protein binding. We detect and annotate condition-specific changes in ΔSHAPE sites under DNA damage, hypoxic stress, and inhibition of cap-dependent translation.

**Figure 1:**
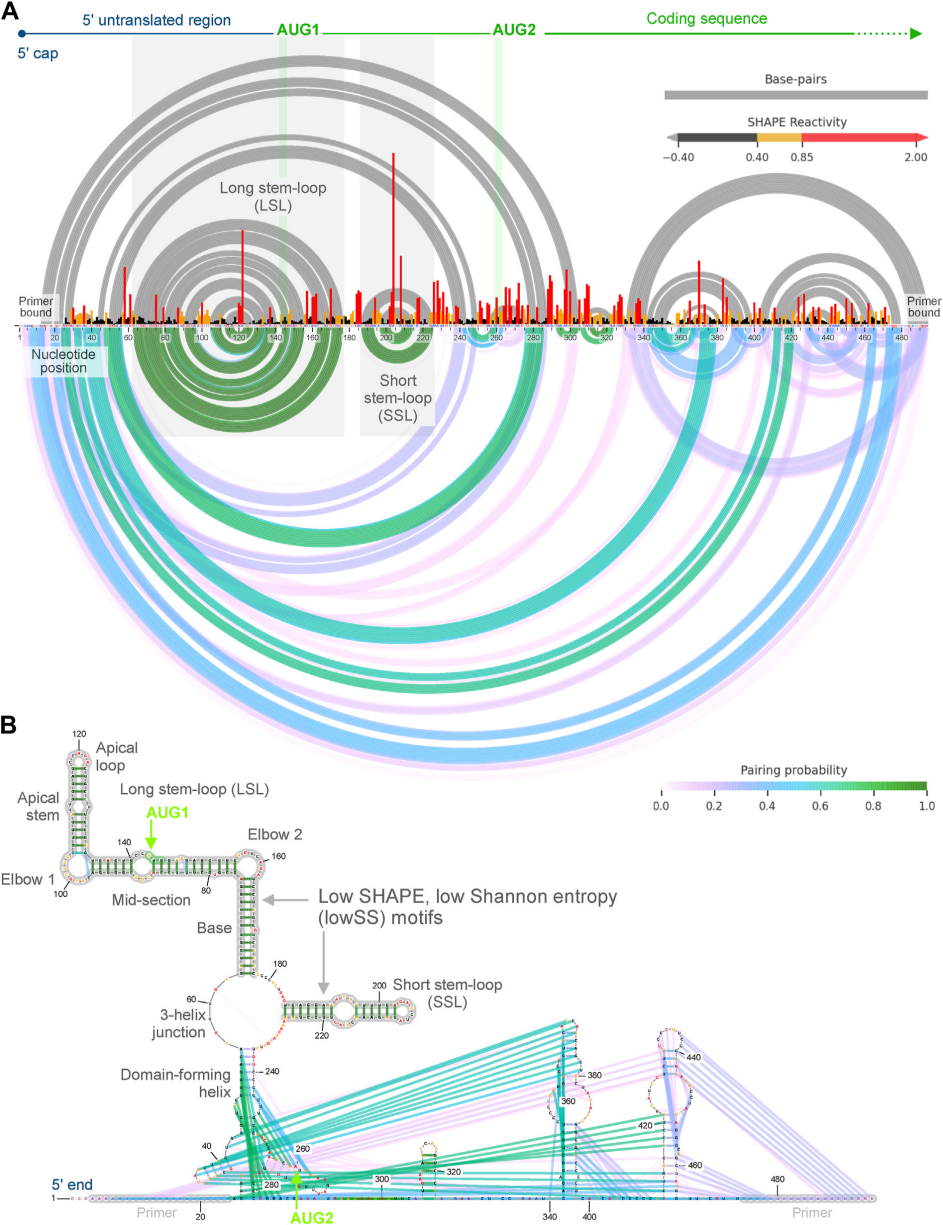
**Cell-free SHAPE reactivity profile, base-pairing probability analysis, and secondary structure model of *TP53* mRNA 5′ end.***A*, normalized reactivity profile using 5-nitroisatoic anhydride (5NIA) SHAPE reagent on gently extracted total RNA from A549 cells. SHAPE profile represents data for nucleotides 1 to 494 aligned to *TP53* mRNA transcript variant 1, NM_000546.6, with transcription start site annotated as nucleotide position 1. Base-pairing is represented using arcs in a color gradient corresponding with pairing probability values. *Gray arcs* represent SHAPE-guided minimum free-energy secondary structure base-pairs. *B*, secondary structure diagram is overlayed with SHAPE reactivity and base-pairing data. Primer bound sites have no SHAPE data and are set as unpaired. *Gray boxes and motifs* outlined in *gray* indicate low SHAPE, low Shannon entropy (lowSS) sites. They also represent two regulatory motifs long-stem loop (LSL, 63–176) and short-stem loop (SSL, 187–225). Start codons AUG1 and AUG2 are shown in *green boxes*. SHAPE, selective 2′ hydroxyl acylation analyzed by primer extension.

## Results

### Cell-free structure model using gently extracted RNA from A549 cells

Applying the SHAPE-MaP short RNA workflow, we measured reactivity to SHAPE reagent 5NIA within the targeted region between positions 25 to 475 in the *TP53* mRNA 5′ end using gently deproteinated RNA extracted from A549 cells and probed under buffer conditions that promote RNA folding (Fig. 1) (32). Primer binding sites flanking these positions have no SHAPE data. Nucleotides 1 to 142 form the 5′ untranslated region for initiation at AUG1 and the coding sequence includes an in-frame AUG2 at position 260-2. Two biological replicates have a Pearson correlation coefficient (r) of 0.97 (Fig. S1*A*). We detect high mutation rate relative to an untreated control (Fig. S2). SHAPE data were applied to guide secondary structure modeling and base-pairing probability calculations. Several positions with pairing probability greater than 80% are within lowSS (SHAPE <0.4 and Shannon entropy <0.08; where Shannon entropy represents degree of uncertainty for base-pairing) motifs, LSL and SSL (shaded in gray) (Fig. 1). We focus most of our analysis specifically on LSL and SSL which have the highest similarity scores for secondary structure between cell-free replicates (Table S1).

**Figure 2:**
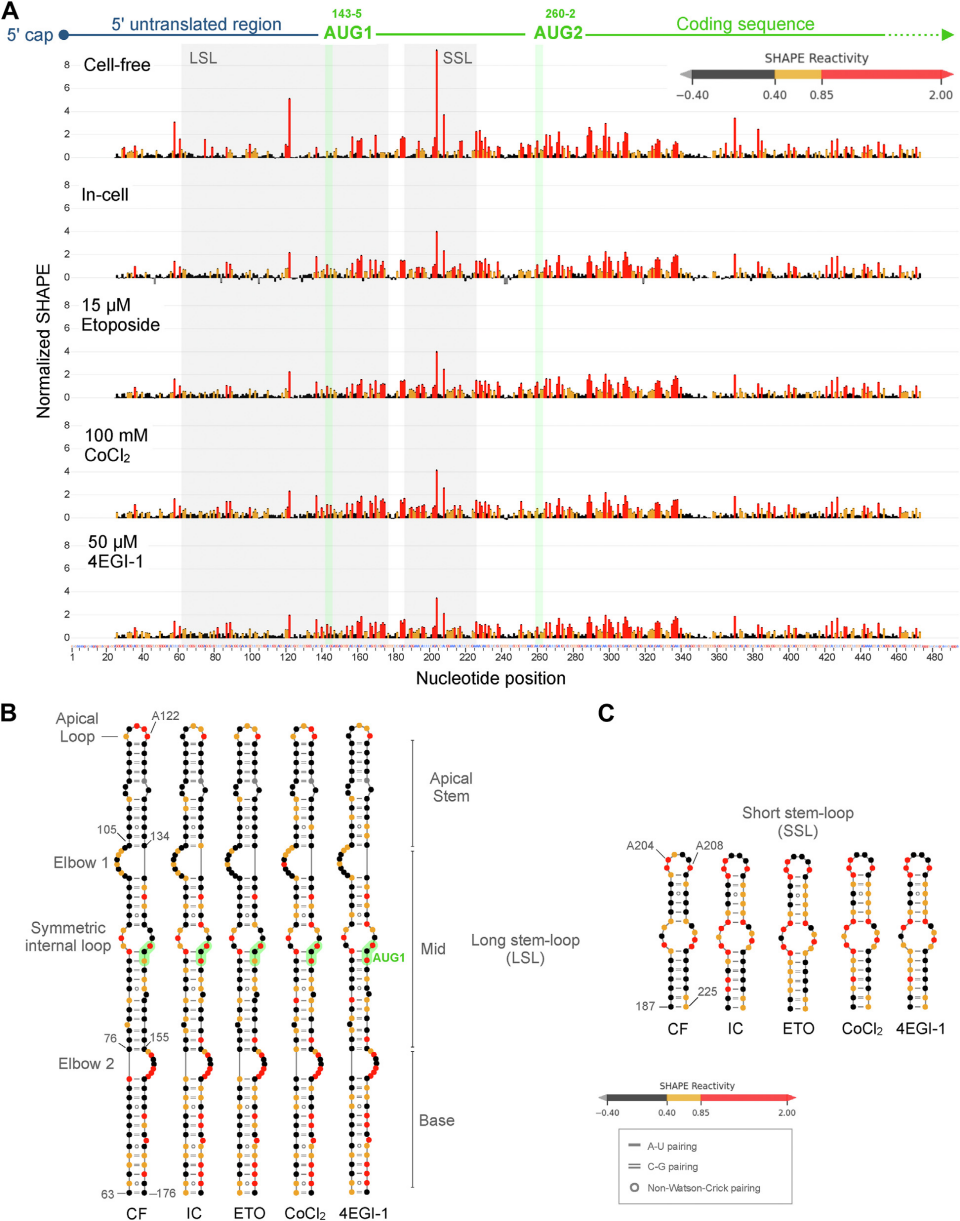
**In-cell SHAPE profiles for all conditions compared to cell-free**. *A*, normalized 5NIA SHAPE reactivity is compared across different in-cell treatment conditions in live A549 cells. Data colored in *red*, *orange*, and *black* indicate high, intermediate, and low SHAPE reactivity, respectively. *B* and *C*, SHAPE data for different conditions are plotted on the cell-free structure model for specific structural features LSL and SSL, respectively. 5NIA, 5-nitroisatoic anhydride; LSL, long stem-loop; SHAPE, selective 2′ hydroxyl acylation analyzed by primer extension; SSL, short stem-loop.

LSL spans 114 nts with the AUG1 start codon located in the mid-section within a 10-member symmetrical internal loop (Fig. 1 and 2). The 7-nucleotide LSL apical loop has three high reactivity positions (Figs. 1*B* and 2*B*). The apical stem positions of LSL have low reactivity nucleotides in nearly all paired positions (Figs. 1*B* and 2*B*). An asymmetric internal loop, with intermediate SHAPE reactivity forms elbow one joining the apical stem to the mid-section (Figs. 1*B* and 2*B*). We observe five intermediate and one high reactivity nucleotide in paired positions along nucleotides 135-48 (Fig. 1*B*). The LSL base is formed by a 13 bp helix featuring three non-Watson–Crick base pairs, with some intermediate and high SHAPE reactivity positions (Figs. 1*B* and 2*B*).

**Figure 3:**
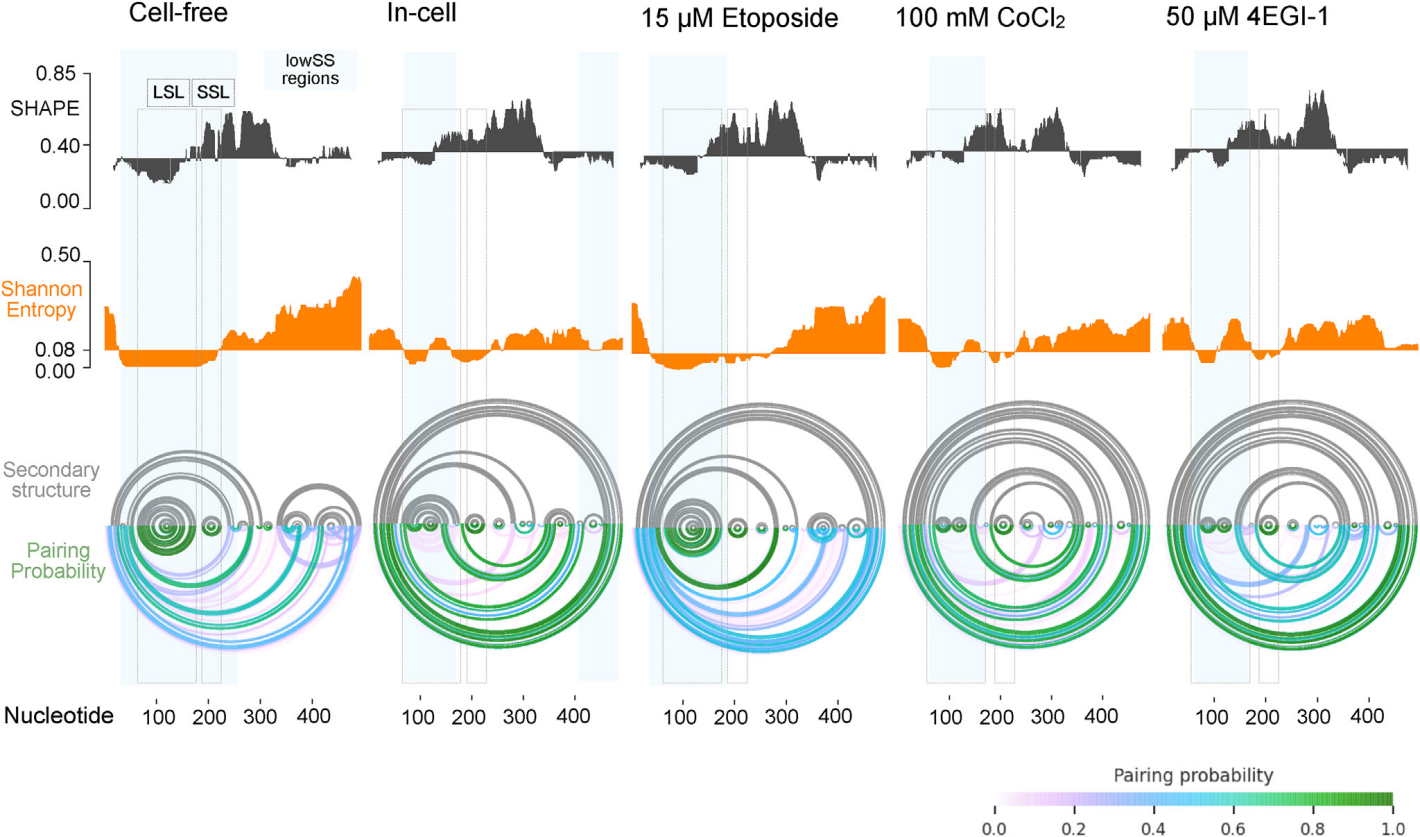
**Comparison of low SHAPE, low Shannon entropy regions, and pairing probability across in-cell conditions**. LowSS analysis identifies regions (*cyan boxes*) of low SHAPE (*black*) and low Shannon entropy (*orange*). LSL and SSL motifs are identified as boxes with *gray dotted lines*. Secondary structure (*gray arcs*) and pairing probability (*green to pink gradient arcs*) plots are shown below for each condition. LSL, long stem-loop; SHAPE, selective 2′ hydroxyl acylation analyzed by primer extension; SSL, short stem-loop.

SSL (40 nts between positions 187–225) is located between the two start codons. SSL has one slightly asymmetrical internal loop (11 nts) with multiple high reactivity positions and an 8-nt apical loop with the highest reactivity positions (A204 and A208) detected along the entire 5′ end (Figs. 1 and 2). Base-paired positions have low SHAPE reactivity. SSL has low Shannon entropy (<0.08), indicative of a stable and potentially functional motif (Figs. 1*B* and 3). High reactivity unpaired positions 184-6 and 226-34 flank the SSL motif on both sides. Downstream of the SSL, the AUG2 start codon appears within a stem-loop, although nucleotides have alternate base-pairing patterns (Fig. 1, *A* and *B*). LSL and SSL are held by a domain-forming helix (within positions 44–56 and 237–47) with a 10-nt symmetric internal loop; however, these positions have ∼20 to 30% pairing probability under cell-free conditions (Figs. 1*B* and 3). A three-helix junction connects LSL, SSL, and the domain-forming helix (Fig. 1*B*). Downstream structures are likely to form alternate folding patterns.

### In-cell SHAPE-MaP under unstressed and stressed cellular conditions

Pearson correlation coefficient values between replicates for in-cell SHAPE reactivity data across in-cell unstressed and chemically stressed conditions are high (Fig. S1*A*). Overall, in-cell SHAPE profiles are consistent in pattern of reactivity peaks (Figs. 2*A* and S1*B*). With 5NIA SHAPE reagent, we detect high mutation rate relative to an untreated control across in-cell unstressed and stressed conditions (Fig. S2). Similarity scores of SHAPE-reactivity–based secondary structures between replicates of each treatment condition show high reproducibility between replicates for all conditions except CoCl_2_ (Table S1). Within the lowSS region, similarity score for CoCl_2_ replicates is higher but relatively low compared to other in-cell conditions. For lowSS nucleotides, very high similarity is observed for unstressed and etoposide stressed cells (Table S1).

Plotted on the cell-free secondary structure motifs LSL and SSL, some differences in the distribution of high and intermediate reactivity positions are noticeable (Fig. 2, *B* and *C*). Noteworthy changes are detected on the LSL apical loop and stem, LSL base, and along SSL paired positions (Fig. 2, *B* and *C*). Under live cell conditions, specific mRNA regions are affected by protein binding and other regulatory interactions which can be detected *via* comparative SHAPE analysis.

### Base-pairing probability across in-cell conditions

Compared to the cell-free reference, we detect some changes in high base-pairing probability regions for the unstressed in-cell condition (Figs. 3 and S3). Shannon entropy increases in the LSL base, narrowing the span of lowSS nucleotides when compared to the cell-free state. LSL apical end and SSL retain high (>80%) pairing probability under all in-cell unstressed and stressed conditions (Figs. 3 and S3). Other positions on LSL mid-section and base show high SHAPE-informed pairing probability with different nucleotides. Etoposide treatment produces high probability pairing along the entire LSL, identical to the cell-free model (Fig. 3). CoCl_2_-treated replicates show variability in LSL base-pairing (Figs. 3 and S4). Intensity of SHAPE reactivity on LSL base positions is higher in replicate 1 compared to replicate 2 (Figs. 3 and S4). Total protein levels of FLp53 and Δ40p53 detected using western blots are similar to unstressed cells, with no statistically significant changes (Fig. S5, *A* and *B*).

4EGI-1 treated cells have LSL pairing probability similar to in-cell and distinct from etoposide (Figs. 3 and S4). Protein levels for both FLp53 and Δ40p53 increase with higher concentrations of 4EGI-1 accompanied by decreasing in levels of GAPDH (Fig. S5, *C* and *D*). A549 cells pretreated with 4EGI-1 and subsequently treated with etoposide result increased accumulation of both FLp53 and Δ40p53 relative to GAPDH (Fig. S5, *C* and *D*). SHAPE-MaP analysis on cells with 4EGI-1 and etoposide results in LSL base-pairing similar to the etoposide-only sample (Fig. S4, *B* and *C*). In-cell SHAPE-informed pairing probability changes are likely due to nucleotide flexibility upon binding with transacting factors under specific conditions rather than alternate RNA folding states. To check if LSL can occupy alternate in-cell structural states, we conducted dimethyl sulfate (DMS)-MaP (detects base-pairing by directly probing nucleobases) under unstressed in-cell conditions and applied the DanceMapper deconvolution algorithm (Fig. S6) (38, 39). DANCE-MaP analysis reveals that LSL nucleotides (amplicon 1) cluster into a singular structural state as described in our cell-free model with no alternate folding is detected (Table S2 and Fig. S6*B*). Therefore, while LSL has one stable folding state, normal and stress-related in-cell interactions likely affect nucleotide-level flexibility as detected by SHAPE. Downstream coding positions in amplicon 2 cluster into two distinct folding states at 78% and 22% distribution potentially representing active (less structured) and inactive (more structured) translation states, respectively (Fig. S6*C*).

### ΔSHAPE reveals in-cell interaction sites

**Figure 4:**
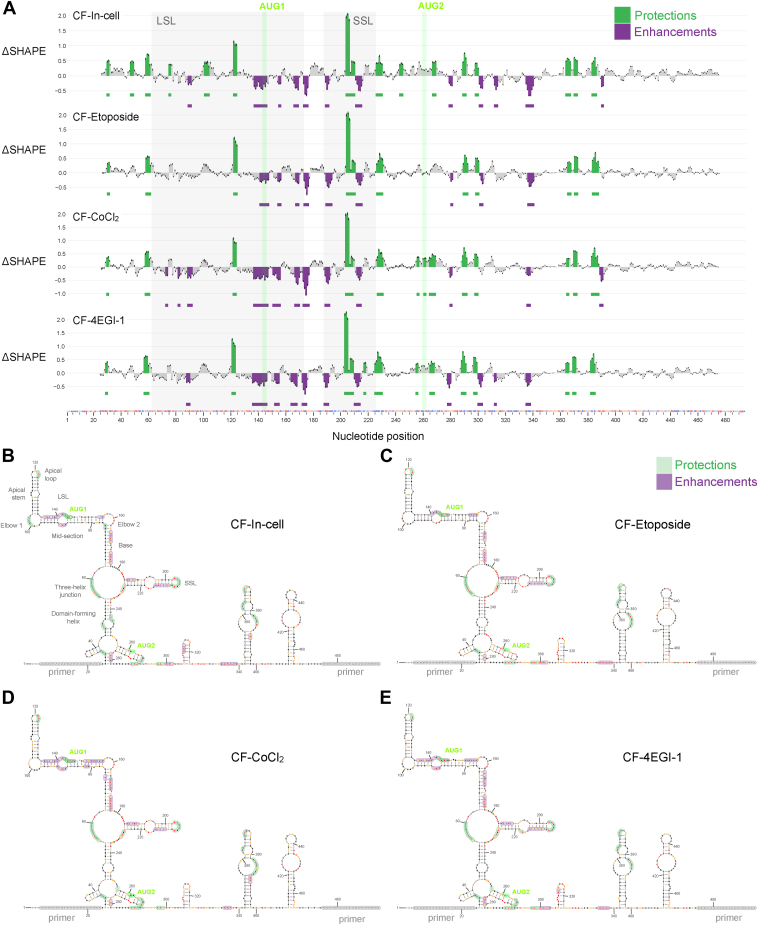
**ΔSHAPE analysis to identify in-cell protections and enhancements across treatment conditions relative to cell-free data**. *A*, relative to cell-free reference data, sites of reduced SHAPE are identified as protections (*green*) while increased SHAPE as enhancements (*purple*). *B–E*, ΔSHAPE enhancement and protection sites along with SHAPE reactivity data for each in-cell unstressed and stressed condition are annotated on the cell-free structure model. SHAPE, selective 2′ hydroxyl acylation analyzed by primer extension.

To methodically assess in-cell changes relative to our cell-free reference data, we apply ΔSHAPE analysis using parameters optimized for detecting in-cell protection and enhancement sites as a result of protein binding (40). Firstly, we identify prominent enhancement sites on the LSL base and mid-section between positions 136-75 (Figs. 4 and S4). Next, within the LSL apical loop, we observe a protection site on positions 121-3 consistently across all in-cell conditions. The asymmetrical internal loop that forms elbow 1 is protected at nucleotides 100-3 only under unstressed conditions (Fig. 4*B*). Furthermore, two protection sites (57–60 and 225–31) are found in all conditions on the 3-helix junction that connects LSL, SSL, and the domain-forming helix (Fig. 4).

On SSL, we observe ΔSHAPE enhancements on opposite ends of helical regions and significant protection on the apical loop. (Fig. 4). The SSL protection site comprises all exposed nucleotides on the apical loop. Protection is also detected on the exposed loop nucleotides 266-8 of the AUG2 containing hairpin, and this ΔSHAPE change is notably absent under etoposide stress (Fig. 4).

Along both sides of the internal loop of the domain-forming helix at positions 46-8 and 242-4, we detect an in-cell protection that is remarkably absent when cells are subjected to stress conditions (Fig. 4). Further downstream, the stem-loop between 342-98 has multiple protections at flexible positions while the stem-loop at 411-65 is unchanged, and these ΔSHAPE changes within coding sequence are consistent under different stress conditions (Fig. 4).

### Stress-induced ΔSHAPE relative to unstressed in-cell condition

**Figure 5:**
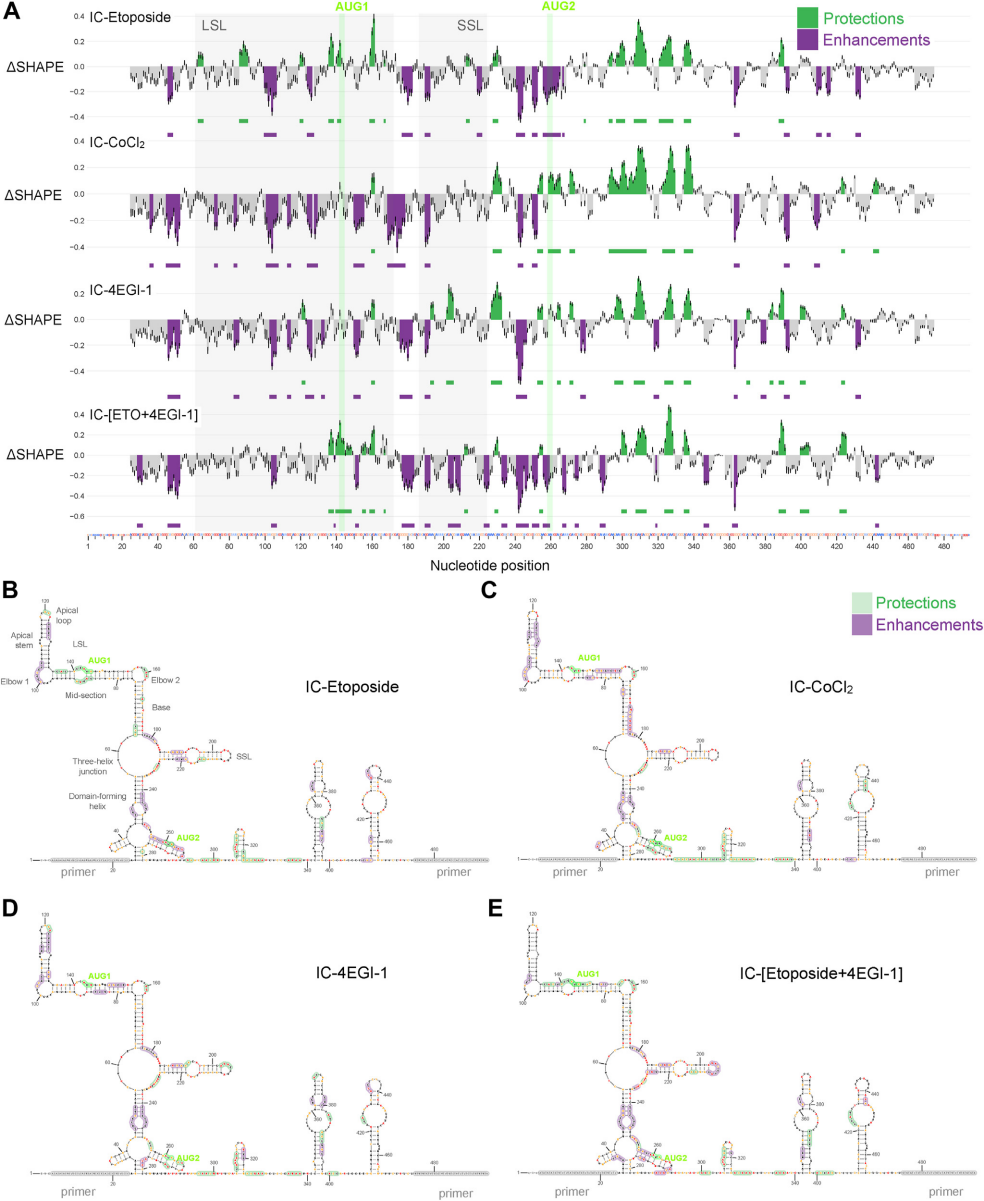
**ΔSHAPE analysis to identify stress-induced protections and enhancements across treatment conditions relative to unstressed in-cell data**. *A*, relative to unstressed in-cell reference data, sites of reduced SHAPE are identified as protections (*green*) while increased SHAPE as enhancements (*purple*). *B*–*E*, ΔSHAPE enhancement and protection sites along with SHAPE reactivity data for each in-cell unstressed and stressed condition are annotated on the cell-free structure model. SHAPE, selective 2′ hydroxyl acylation analyzed by primer extension.

**Figure 6:**
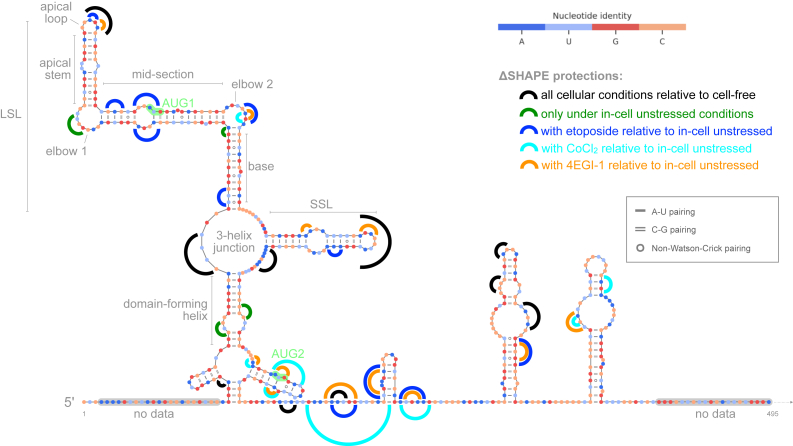
**ΔSHAPE protections in different cellular conditions relative to cell-free or in-cell data.** Protections common to all unstressed and stressed in-cell conditions relative to cell-free are shown in *black*. Protections detected only in unstressed cells are shown in *green*. Stress-specific protections relative to unstressed cells are shown in *dark blue* (etoposide only), *cyan* (CoCl_2_ only), and *orange* (4EGI-1 only). SHAPE, selective 2′ hydroxyl acylation analyzed by primer extension.

Using the unstressed in-cell SHAPE data as a reference, we analyzed relative effects of cellular stress conditions with ΔSHAPE (Fig. 5). Overall, SHAPE reactivity is higher under stress compared to unstressed cells, and several sites of ΔSHAPE enhancements are detected particularly within the first 300 positions of the *TP53* mRNA 5′ end (Fig. 5*A*). A striking stress-associated change is the enhancement (or absence of protection) at the domain-forming helix and its internal loop, observed consistently under all stress conditions (Fig. 5, *B*–*E*). Enhancements also appear in all stress conditions on the LSL apical stem and elbow 1 and along certain SSL paired positions (Fig. 5, *B*–*E*). Consistent stress-associated protection is detected on elbow 2 and along nucleotides 300 to 340 (Figs. 5 and 6).

Some ΔSHAPE sites are dependent on the type of stress. Under etoposide stress, protections in the LSL mid-section are noteworthy (Fig. 5*B*). This observation is sustained under the combined effect of etoposide and 4EGI-1; however, protections only appear along the AUG1 side of LSL (Fig. 5*E*). Protections associated with both CoCl_2_ and 4EGI-1 are observed near AUG2 and with 4EGI-1 alone on SSL (Fig. 5, *C* and *D*). Some noteworthy stress-associated enhancements include etoposide-induced enhancements around AUG2 (Fig. 5*B*), CoCl_2_- and 4EGI-1- (Fig. 5, *C* and *D*) associated enhancements along LSL mid-section, and CoCl_2_-induced enhancement on the LSL base. The combined effect of etoposide and 4EGI-1 is similar to the etoposide-only sample; however, it has an additional enhancement on SSL apical loop (Fig. 5*E*).

## Discussion

The *TP53* mRNA has been shown to engage in cap-independent translation initiation (10, 11, 12). The role of RNA motifs, transacting factors, and increased p53 levels in response to stress is relatively well-established (1, 9). Prior approaches have used human and mouse variant constructs for *in vitro* analysis and transfected constructs for *ex vivo* and in-cell analysis (15, 41). Structural studies on endogenous *TP53* mRNA in its cellular context remain a critical gap in this field. Several details of the mechanism by which *TP53* switches from cap-dependent to cap-independent translation initiation under stress still need to be elucidated. Our work applies comparative SHAPE-MaP analysis on previously described *TP53* mRNA 5′ end motifs under selected cellular stress conditions associated with cap-independent initiation.

We apply the SHAPE-MaP chemical probing strategy to describe the endogenous *TP53* mRNA 5′ end structure, along with comparative analysis of localized changes in per-nucleotide SHAPE reactivity (34, 40). We use 5NIA as the SHAPE reagent, previously shown to be advantageous for in-cell analysis in mammalian cells due to its high membrane penetrability, relatively short half-life, and high signal-to-noise (37, 42). In-cell SHAPE-MaP analysis requires a cell-free reference model from extracted and deproteinated RNA (Fig. 1), treated with the same SHAPE reagent in order to calculate differences in the per-nucleotide reactivity profile (22, 28). In-cell SHAPE and pairing data are meaningful when analyzed in a comparative context relative to a cell-free model since in-cell interactions, active mRNA translation, and ensemble mRNA structural states may influence in-cell data.

Our data show that LSL can exist in its fully structured state in live cells with some indication of in-cell flexibility along positions on the LSL base in response to cellular interactions (Figs. 2*B* and 3). The LSL apical end remains at high pairing probability under all conditions (Figs. 1*B* and 2*B*, and S3). SHAPE reagents probe the 2ʹOH group of the ribose sugar rather than the nucleobase and therefore are sensitive to localized nucleotide flexibility caused by a number of factors including cellular RNA–protein interactions. Nucleotide flexibility observed on the LSL base is expected for AUG1-mediated translation (Fig. 2*B*); however, our SHAPE data do not indicate in-cell refolding into an alternate stable structure. Base-probing with DMS-MaP analyzed using deconvolution algorithms like DanceMapper can reveal alternate in-cell folding states (38, 39). With DANCE-MaP, we find that LSL and SSL motifs do not fold into alternate structures under normal in-cell conditions (Fig. S6*B*). Downstream nucleotides located between 247 and 493 cluster into two structural states at 78% and 22%, respectively where the relatively unstructured state 1 likely represents a translationally active condition (Fig. S6*C*).

A ΔSHAPE protection site is detected on the LSL apical loop, and this protection is enhanced with etoposide and 4EGI-1 treatment (Figs. 4, 5, *B*, *D* and *E*, and 6). This protection is potentially due to reported interaction with transacting factors like PTB and Ku (18, 43). The LSL motif, particularly the LSL apical end, has been reported to play a critical role in the cap-independent translation mechanism based on prior studies using reporter assays and mutational analysis (8, 9, 11, 16, 20). We see some stress-associated ΔSHAPE enhancements along the LSL apical stem suggestive of changes that may result from protein binding to the apical loop (Fig. 5, *B*–*E*). The LSL apical end is likely to reform after momentary binding-induced changes since despite stress-induced enhancements, the apical end maintains high pairing probability in all cellular conditions consistent with the cell-free model (Figs. 1 and 3). The LSL elbow 1 is protected under unstressed in-cell conditions, and this protection appears to be lost under stress (Figs. 4, *B*–*E* and 6). LSL mid-section contains the AUG1 codon, and we observe etoposide-stress associated ΔSHAPE protections on both sides of the internal loop in this region (Figs. 5*B* and 6). With combined etoposide and 4EGI-1 treatment, the ΔSHAPE protection favors the AUG1 side of LSL (Fig. 5*E*). Other prominent etoposide-linked protections are detected on LSL elbow 2 and base (Fig. 5*B*). Perhaps the LSL mid-section protection is indicative of binding by a DNA damage-induced transacting factor or a result of active translation using AUG1.

SSL has high base-pairing probability across in-cell and stress conditions (Fig. 3). The SSL motif is a known binding site for transacting factors HDM2/HDMX, Annexin A2, PSF, PTB, and hnRNP C1/C2 (6, 16, 21, 44). Protein binding with transacting factors likely results in ΔSHAPE protections observed at exposed positions on and around SSL, specifically at nucleotides 203-8 of the apical loop, 217-8 on the internal loop, and at SSL adjacent 225-30 (Fig. 4) (40). Consistent enhancements along paired positions on SSL helices suggest in-cell unpairing (Fig. 4, *B*–*E*). Further enhancement is detected on the SSL lower stem positions under cellular stress (Fig. 5, *B*–*E*). SSL unpairing may be necessary for codon access during AUG1 translation; however, the persistently high pairing probability suggests that SSL is likely to spring back into its stem-loop conformation after momentary unwinding (Figs. 3 and S3). With cap-inhibitor treatment, we observe additional protection on SSL; however, in combination with etoposide, the protection is lost, and the SSL apical loop is more reactive compared to in-cell (Fig. 5, *D* and *E*). Potentially, protein binding at the SSL apical loop is relatively lower with DNA damage as compared to 4EGI-1 treated cells, and in combination with 4EGI-1, the influence of DNA damage is more pronounced (Fig. 5, *D* and *E*).

The three-helix junction has in-cell protection under all cellular conditions (Figs. 4, *B*–*E* and 6). In contrast, the domain-forming helix is protected on both sides of the helix only in unstressed cells (Figs. 4*B*, 5, *B*–*E*, and 6). Protein interaction at the domain-forming helix maybe disengaged under stress (Fig. 6). The short hairpin containing AUG2 is protected at its exposed loop in all conditions except under DNA damage (Figs. 4, *B*–*E*, and 6). Relative to unstressed cells, while hypoxia and cap-inhibition cells have protections near AUG2, DNA damage results in increased reactivity along nucleotides in this region (Figs. 5, *B*–*E* and 6). It is possible that an AUG2-proximal binding factor is inactive under genotoxic stress.

In summary, our investigation reveals several stress-associated interaction sites that may be critical to the mechanism of cap-independent initiation of *TP53* in live human A549 cells (Fig. 6). We can now better contextualize previous reports on IRES activity in *TP53*, and we provide a framework to extend this analysis to other mRNAs that putatively engage in noncanonical translation initiation (14, 45). Future studies on *TP53* mRNA applying single-molecule correlated chemical probing under different conditions may help describe a potential regulatory role of through-space RNA–RNA interactions (38, 46). Critically, precise identification of mRNA-binding proteins and elucidating their interaction sites *via* crosslinking-based strategies will further refine our description of stress-associated interaction sites on the *TP53* 5′ regulatory region (47).

## Experimental procedures

### Cell culture

A549 human lung carcinoma cells (ATCC, catalog no. CCL-185) were maintained in HyClone Dulbecco's Modified Eagles Medium (Cytiva, catalog no. SH3008101) and supplemented with 10% FCS (Cytiva, catalog no. SH3007203) and 1% penicillin-streptomycin glutamine (100×) (Gibco, catalog no. 10378016). Cells were grown in 5% CO_2_ on standard 10 cm tissue culture plates or T75 flasks and utilized before cells were completely confluent. Cells were routinely tested for *mycoplasma* contamination using PCR-based detection (ATCC, 136-XV).

### Cell-free RNA extraction and SHAPE treatment with 5NIA

For cell-free sample preparation, RNA was gently extracted using a previously described procedure optimized for native RNA folding (37). A549 cells around 90% confluence were washed and pelleted in PBS (Cytiva, catalog no. SH3025601) then resuspended in 2.5 ml lysis buffer [40 mM Tris-HCl (pH 8.0), 25 mM NaCl, 6 mM MgCl_2_, 1 mM CaCl_2_, 256 mM sucrose, 0.5% Triton X-100] and incubated at room temperature for 5 min. Cells in lysis buffer were split equally into five microcentrifuge tubes. One volume of phenol/chloroform/isoamyl alcohol saturated with 100 mM Tris-EDTA to pH 8.0 (24:24:1, Thermo Scientific, catalog no. AC327111000) was added to each sample then inverted several times and centrifuged at 12,000*g* for 15 min at 4 °C. This was repeated for a total of two extractions. Then with one volume or 500 μl of pure chloroform (Thermo Scientific, catalog no. AC158210010), the same was done two more times to remove excess phenol. The final aqueous layer was exchanged into 1.1× folding buffer [111 mM Hepes (pH 8.0), 111 mM NaCl, and 5.55 mM MgCl2] using a PD-10 desalting column (Cytiva catalog no. 17085101) and incubated at 37 °C for 30 min, and 3.5 ml of the buffer-exchanged sample was split into two equal volumes and collected in 15 ml conical tubes. For one 15 ml conical tube, 1/9 of the volume of 250 mM 5NIA (CAS No. 4693-02-1; AstaTech Inc, catalog no. 69445) in anhydrous dimethyl sulfoxide (DMSO, Thermo Scientific, catalog no. AA43998AE) was added. A no-reagent negative control was prepared using the other conical similarly, with neat DMSO instead of 5NIA. Both 5NIA treated and untreated samples were incubated at 37 °C for 10 min, and 1/25 volume of 5 M NaCl and one volume isopropanol were then added to each tube. Samples were incubated at room temperature for 10 min to precipitate RNA. After 10 min of centrifugation at 10,000*g* at room temperature, pellets were washed with 75% ethanol and spun down again at 7500*g* for an additional 5 min at room temperature. Fifty milliliter of 1× TURBO DNase buffer was used to resuspend the pellet for each sample along with 1 μl TURBO DNase enzyme (Invitrogen, catalog no. AM2238). DNase treatment was carried out at 37 °C for 1 hour. RNA was purified with 1.8× ratio Mag-Bind Total Pure NGS (Omega Bio-Tek Inc, catalog no. NC1545573) magnetic bead clean up and eluted in approximately 30 μl of nuclease-free water.

### In-cell SHAPE modification and RNA extraction

A549 cells were grown to 80 to 90% confluence in 10 cm plates or T75 flasks for untreated and SHAPE-treated samples. After aspirating growth medium and a PBS wash, equal volume of fresh media was added to adherent cells. Cells were resuspended using a cell scraper and transferred to two 15 ml conical tubes. For in-cell SHAPE probing, 1 ml of 250 mM 5NIA in DMSO (final concentration of 25 mM) was added to the modified sample. The untreated control cells were treated with 1 ml DMSO. Cells were incubated at 37 °C for 15 min after which samples were split into microcentrifuge tubes for centrifugation at 7500*g* for 5 min at room temperature. For total RNA extraction, 1 ml TRIzol (Invitrogen, catalog no. 15-596-026) was added to each pellet and mixed, pooling each pellet into a single tube for 5 min of room temperature incubation. Next, 200 μl of pure chloroform was added, and samples were gently mixed by inverting tubes and then incubated for 5 min at room temperature and centrifuged at 12,000*g* for 15 min at 4 °C. The aqueous layer was transferred to clean tubes to which 500 μl isopropanol (Fisher Scientific, catalog no. BP26181) was added and incubated for 10 min at room temperature. Samples were centrifuged at 12,000*g* for 10 min at 4 °C. Pellets were washed with 1 ml 75% ethanol then treated with TURBO DNase and purified similarly as described above for cell-free RNA extraction.

### Treatment with stress inducers and cap-inhibitor

Genotoxic and hypoxic stress were induced chemically using etoposide (CAS no. 33419420, Sigma-Aldrich catalog no. E1383–100 MG) and cobalt chloride hexahydrate (CoCl_2_, CAS no. 7791-13-1, MP Biomedicals), respectively. Etoposide treatment using 15 μM (in DMSO) final concentration was done at 80% confluence, after which cells were allowed to grow for 12 h before SHAPE treatment and RNA extraction as described for the in-cell condition. For hypoxia treatment, a final concentration of 100 mM CoCl_2_ (in DMSO) was added to cells at the point of subculturing cells onto a fresh plate. Cells were allowed to grow up to 80 to 90% confluence before carrying out standard in-cell SHAPE workflow. To inhibit cap-dependent initiation, we used eIF4E/eIF4G interaction inhibitor, 4EGI-1 (Sigma-Aldrich, catalog no. 324519) at a final concentration of 50 μM for SHAPE analysis and at 25, 50, and 100 μM when preparing lysates for Western blotting. 4EGI-1 was added to cells at 50 to 60% confluence, and cells were allowed to grow for 24 h prior to either in-cell SHAPE workflow or lysate preparation for western blots. To test the combined effect of cap inhibition and etoposide, cells at 50 to 60% were first treated with 4EGI-1 at the specified concentration for 12 h, then treated with 15 μM etoposide, and grown for an additional 12 h at which point cells were at 85 to 90% confluence. This was followed by lysate preparation or in-cell SHAPE treatment.

### Reverse transcription and library preparation using 2-step PCR

For each cell-free or in-cell sample, cDNA synthesis was carried out with a target-specific reverse transcription primer (Table S2) following previously described MaP conditions (32, 34, 35, 46). 10× MaP prebuffer (500 mM Tris pH 8.0, 750 mM KCl, 100 mM DTT) was prepared in a nuclease-free microcentrifuge tube. Approximately 3 μg of either untreated or SHAPE-modified RNA was preincubated in a PCR tube along with 2 μmole reverse transcription primer, 10 mM dNTP mix, and nuclease-free water up to total volume of 10 μl at 65 °C for 5 min followed by 4 °C for 2 min. A reaction master mix (1×, 2 μl of 10× MaP prebuffer, 1.8 μl nuclease-free water, 4 μl of 5 M Betaine and 1.2 μl of 100 mM MnCl_2_) was freshly prepared and added to the pre-incubated RNA. Sample was mixed well by gentle pipetting and incubated at 42 °C for 5 min in a thermal cycler. Next, 1 μl of SuperScript II (Invitrogen, catalog no. 18-064-014) reverse transcriptase was added to the reaction tube, mixed by pipetting, and incubated in a thermal cycler at 42 °C for 180 min before heat inactivation at 70 °C for 10 min cDNA was purified using Mag-Bind Total Pure NGS bead clean-up.

Five microliter of each cDNA sample was used in a 50 μl step 1 PCR reaction with 1× Q5 reaction buffer, forward and reverse primers at 500 nM, 200 μM dNTPs, and 0.02 unit/μl Q5 Hot-Start High-Fidelity DNA polymerase (New England Biolabs catalog no. M0493L). Step 1 primers also add adapters to the PCR product (Table S2). The reaction condition was 98 °C for 30 s, 20 cycles of 98 °C for 10 s, 67 °C for 30 s, 72 °C for 20 s, and then 72 °C for 2 min. Step 1 PCR products were purified using 1.8× Mag-Bind bead clean-up and eluted in 20 μl nuclease-free water. PCR step 2 was run in order to add TruSeq DNA Single Indexes Set A and B (Illumina, catalog No. 200015964/5) to DNA libraries for multiplexed NGS. Using 10 μl step 1 purified product as template, step 2 PCR reaction had 1× Q5 reaction buffer, 500 nM TruSeq index primers, 200 μM dNTPs, and 0.002 unit/μl Q5 Hot-Start High-Fidelity DNA polymerase. Step 2 PCR conditions were as follows: 98 °C for 10 s, 65 °C for 30 s, 72 °C for 20 s, and then 72 °C for 2 min. Step 2 products were purified with Mag-Bind bead clean up and eluted in 20 μl nuclease-free water. Libraries were quantitated fluorometrically using Invitrogen Qubit 4 and assessed for quality on Agilent TapeStation 4200 using DNA High Sensitivity D1000 reagents (Agilent, catalog no. 5067-5584/5).

### Illumina sequencing and analysis with ShapeMapper2

Purified, high-quality libraries were pooled and sequenced on an Illumina iSeq 100 instrument generating 2 × 150 paired-end data sets. FASTQ files from sequencing runs were input for analysis with *ShapeMapper2* (35). *ShapeMapper2* was run locally on a System76 Thelio Mega Linux workstation and applied the --primers parameter to merge overlapping amplicons. Minimum read depth was set at 5000 for untreated and modified samples. SHAPE reactivity profiles, including error estimates, were created by aligning reads to *TP53* mRNA transcript variant 1 reference sequence (NCBI Reference Sequence accession number NM_000546.6). Median per-nucleotide read depths were greater than 50,000 for both untreated and SHAPE-modified samples.

### Data analysis, structure modeling, and visualization

Linear regression analysis of SHAPE reactivity data between replicates was used to calculate Pearson correlation coefficient (r). SHAPE data files (Supplementary materials) contain per-nucleotide standard error which are also plotted on SHAPE reactivity profiles (Figs. 1*A* and 2*A*, and S1*B*). Secondary structure models and base-pairing probability calculation for the *TP53* 5′ end were generated using SuperFold, which is based on the RNAstructure package (33, 48). Structure information in the merged connectivity table output file was used to visualize and adjust the layout for the secondary structure using VARNA and then exported as a .VARNA session file (49). Base-pairing probability data using the merged dot plot/probability file was used as input for visualizing pairing probability. Sensitivity and positive predictive value calculations used the RNAstructure package *scorer* tool (48). SHAPE profile and secondary structure data visualization, including overlay of SHAPE and pairing probability data on the secondary structure was conducted using RNAvigate (50). ΔSHAPE analysis and plots were also generated using RNAvigate using parameters optimized to detect in-cell interactions (smoothing_window=3, zf_coeff=1.96, ss_thresh= 1, site_window=3, site_nts=2) (40, 50).

### In-cell DMS-MaP and Dance-MaP deconvolution

A549 cells were treated with DMS (Sigma-Aldrich, catalog no. D186309) alongside an untreated ethanol control as previously described (38, 39). Briefly, once cells reached 75% confluency in 10 cm plates, fresh media with 1M bicine was added to the treated plate. Cells were allowed to equilibrate for 3 min at room temperature. 1.7 M DMS in ethanol was then added, and cells were incubated at 37 °C for 6 min. The reaction was quenched with 20% 2-mercaptoethanol (diluted in ice-cold PBS; Thermo Scientific, catalog no. 125472500). The cells were scraped and centrifuged for 5 min at 4 °C and 1000*g*. Molecular grade neat ethanol was added to the untreated sample. The beforementioned TRIzol in-cell RNA extraction protocol was followed for RNA extracted of untreated and DMS-treated samples. Libraries were prepared as previously described for paired-end sequencing on Illumina NextSeq 2000 platform (GGBC, RRID:SCR_010994).

Dance-MaP analysis was conducted using *DanceMapper,* version 1.1, using default parameters and input files generated from *ShapeMapper2* using *--output-parsed-mutations* option. Overlapping amplicons 1 and 2 are analyzed separately, a requirement for DanceMapper, and no data regions of the total target are included for visualization.

### Western blotting

Cells were grown to 80 to 90% confluence and washed twice with ice-cold PBS. Cells were then lysed using 1 ml RIPA buffer (Thermo Scientific, catalog no. 89900) per 10^6^ cells on ice for 5 min with intermittent manual shaking. Lysates were spun at 15,000*g* for 15 min at room temperature, and the supernatant was collected. Lysates were mixed with NuPAGE LDS Sample Buffer 4× (Invitrogen, catalog no. NP0007) and NuPAGE Reducing Agent 10× (Invitrogen, catalog no. NP0009) and heated at 70 °C for 10 min. The NuPAGE Bis-Tris 4 to 12% protein gel (Invitrogen, catalog no. NP0321BOX) was run in reduced NuPAGE MES SDS Running Buffer (Invitrogen, catalog no. NP0002) at 200V for 35 min. Membrane transfer was conducted using the Invitrogen iBlot3 system with low molecular weight parameters using nitrocellulose transfer stacks (Invitrogen, catalog no. IB33002). Using the iBind Flex system, primary and secondary antibodies (p53, Invitrogen catalog no. MA5-14516 from; GAPDH, Invitrogen catalog no. MA5-44678; HRP, Invitrogen catalog no. 31460) were incubated for 2.5 h. SuperSignal West Atto and Pico Ultimate Sensitivity Substrate (Thermo Scientific, A38554, 34580) were used for chemiluminescent visualization on an Invitrogen iBright system.

## Data availability

Data are provided in supporting information. FASTQ sequencing files are available at NCBI SRA using accession number PRJNA1203256.

## Supporting information

This article contains supporting information.

## Conflict of interest

The authors declare that they have no conflicts of interest with the contents of this article.

## Supporting information

Table S3

TP53 Data
