# Supplementary material for: Stress-induced changes in endogenous TP53 mRNA 5′ regulatory region
Source: J Biol Chem. 2025 Mar 18;301(4):108418. doi: 10.1016/j.jbc.2025.108418 (PMC12018109; doi:10.1016/j.jbc.2025.108418)
Supplement: Figure S1 [file mmc1.pdf]

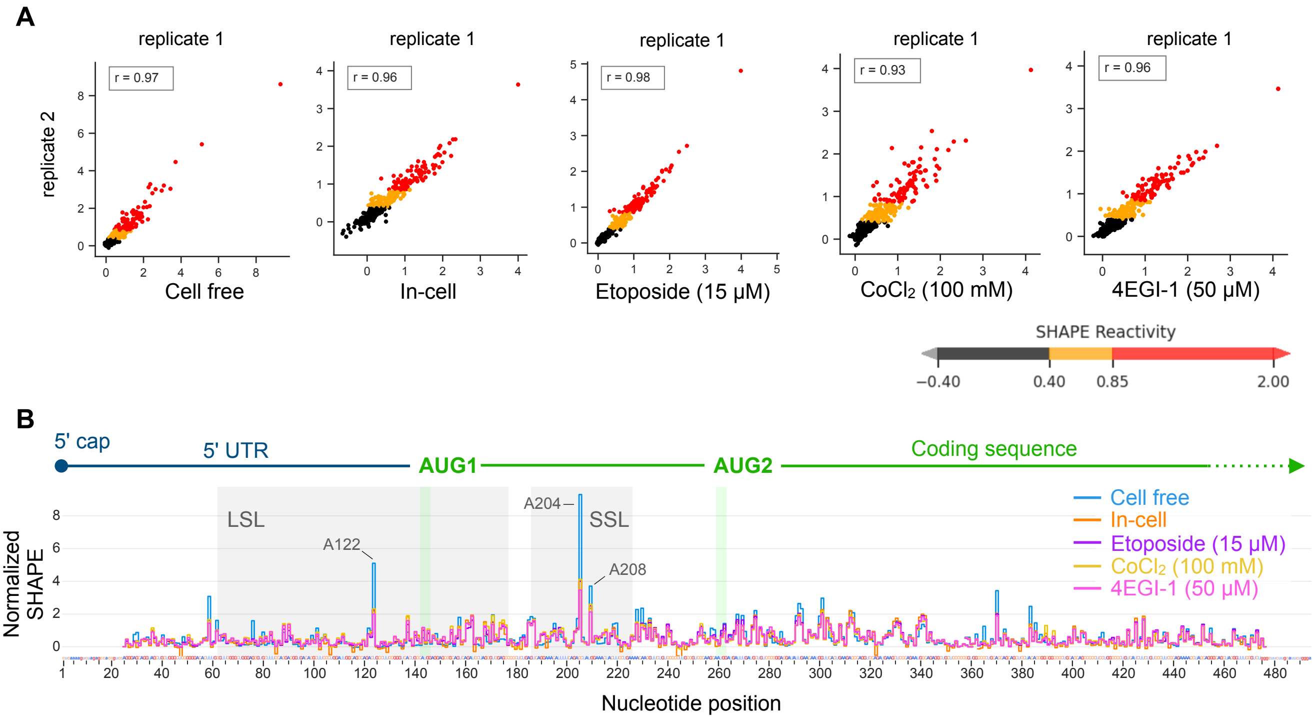

Figure S1. SHAPE reactivity data under cell-free, in-cell unstressed and stressed conditions. (A) Pearson linear regression plots to compare replicate per-nucleotide SHAPE data. Correlation coefficient (r) values are indicated for each analysis. Nucleotides are colored by per-nucleotide SHAPE reactivity. In-cell (IC) sample represents typical A549 cells in absence of added stress. Etoposide (ETO, 15 μM) was added to cells prior to SHAPE treatment to induce genotoxic stress. Cobalt chloride ($CoCl_2$, 100 mM) was added to induce hypoxia. Cap-inhibitor (4EGI-1, 50 μM) was added to block cap-dependent translation. (B) Normalized SHAPE reactivity is compared across different in-cell treatment conditions in live A549 cells and plotted alongside cell-free SHAPE reactivity for comparison.
