# Supplementary material for: Stress-induced changes in endogenous TP53 mRNA 5′ regulatory region
Source: J Biol Chem. 2025 Mar 18;301(4):108418. doi: 10.1016/j.jbc.2025.108418 (PMC12018109; doi:10.1016/j.jbc.2025.108418)
Supplement: Figure S2 [file mmc2.pdf]

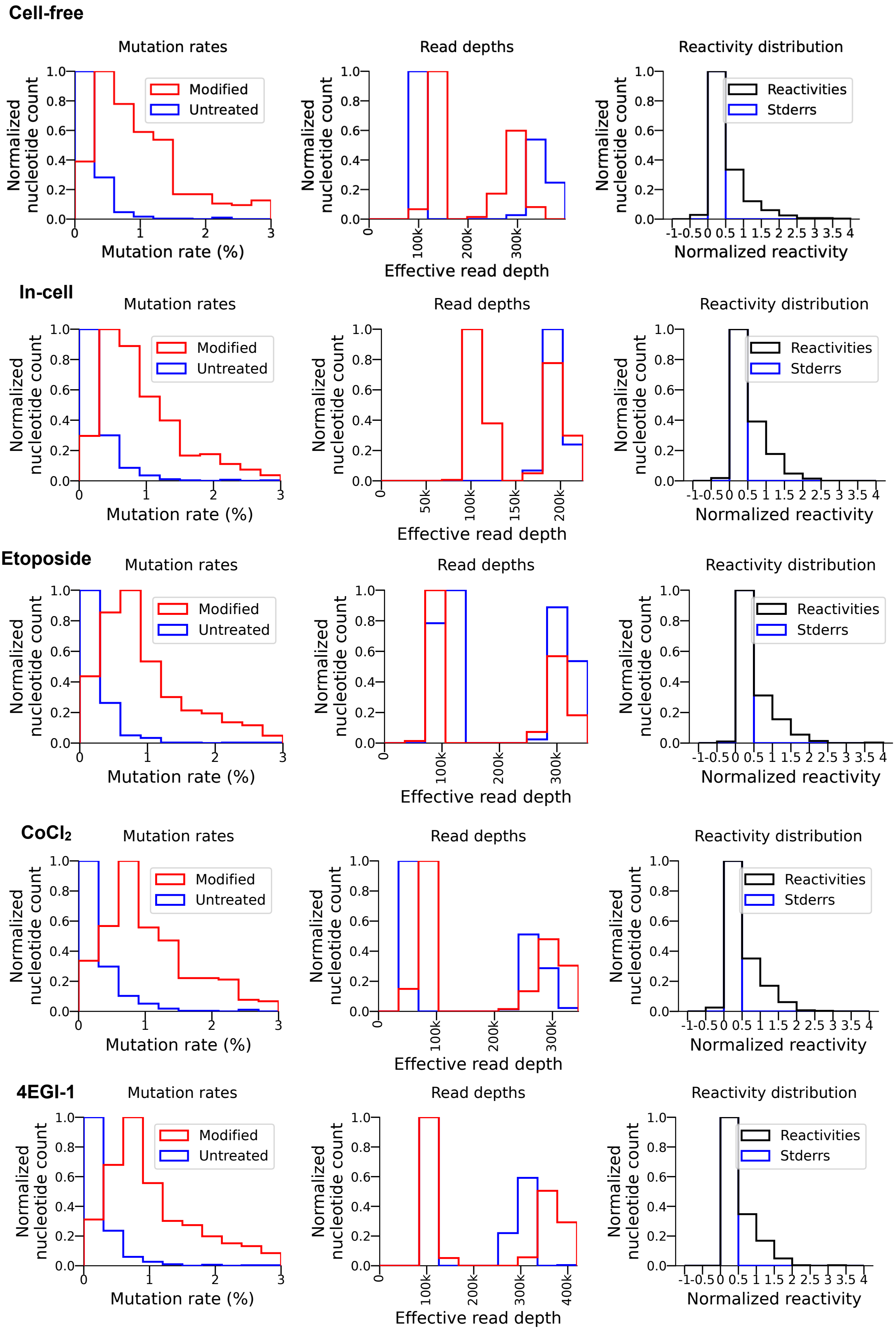
Cell-free
Mutation rates
Read depths
Reactivity distribution
Modified
Untreated
Reactivities
Stderrs
Normalized nucleotide count
Mutation rate (%)
Effective read depth
Normalized reactivity
In-cell
Mutation rates
Read depths
Reactivity distribution
Modified
Untreated
Reactivities
Stderrs
Normalized nucleotide count
Mutation rate (%)
Effective read depth
Normalized reactivity
Etoposide
Mutation rates
Read depths
Reactivity distribution
Modified
Untreated
Reactivities
Stderrs
Normalized nucleotide count
Mutation rate (%)
Effective read depth
Normalized reactivity
CoCl2
Mutation rates
Read depths
Reactivity distribution
Modified
Untreated
Reactivities
Stderrs
Normalized nucleotide count
Mutation rate (%)
Effective read depth
Normalized reactivity
4EGI-1
Mutation rates
Read depths
Reactivity distribution
Modified
Untreated
Reactivities
Stderrs
Normalized nucleotide count
Mutation rate (%)
Effective read depth
Normalized reactivity

Figure S2. SHAPE-MaP histograms representing mutation rate, read depth, and reactivity distribution across cell-free and in-cell conditions.
