# Supplementary material for: Stress-induced changes in endogenous TP53 mRNA 5′ regulatory region
Source: J Biol Chem. 2025 Mar 18;301(4):108418. doi: 10.1016/j.jbc.2025.108418 (PMC12018109; doi:10.1016/j.jbc.2025.108418)
Supplement: Figure S3 [file mmc3.pdf]

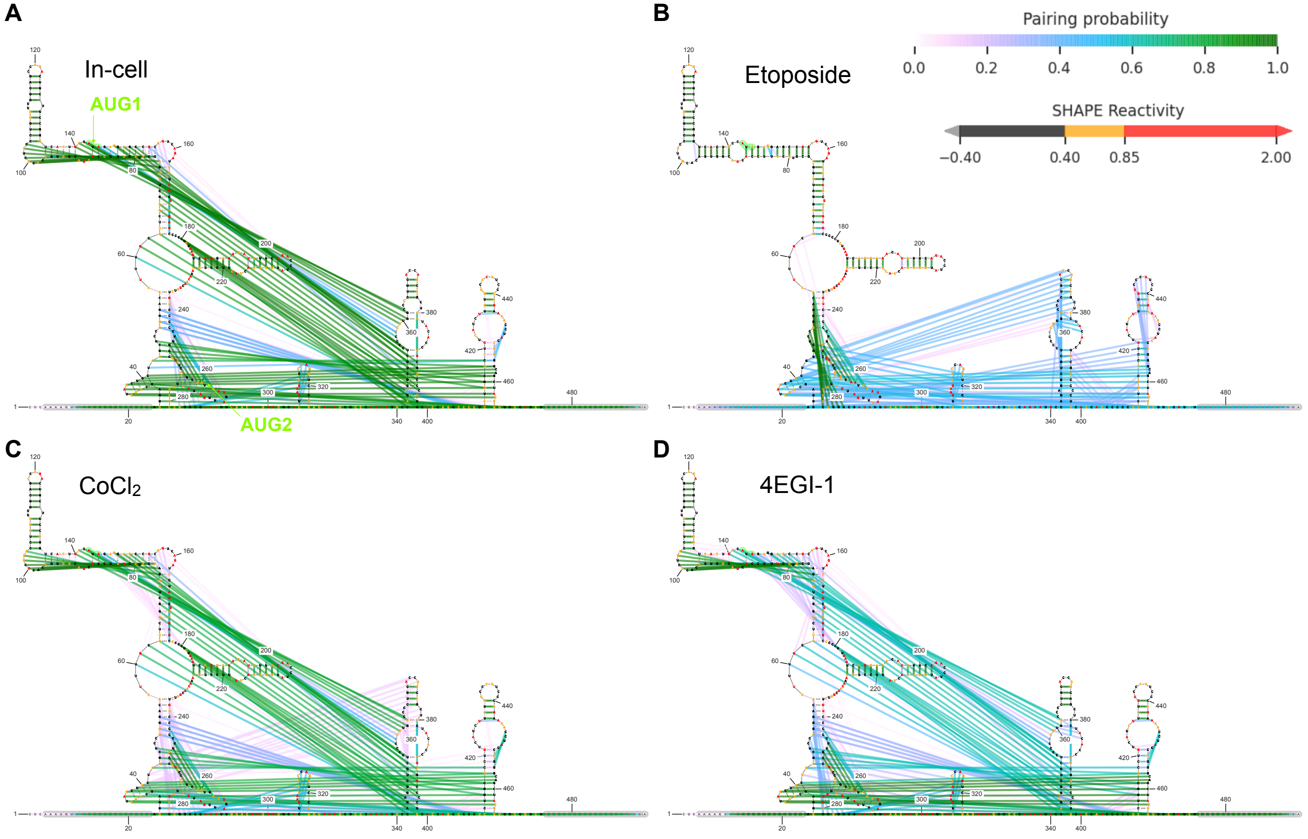

Figure S3. Base-pairing probability data plotted on secondary structure from cell-free condition. (A-D) Base-pairing probability interactions and SHAPE reactivity data (green to pink gradient lines) for each in-cell unstressed and stressed condition are plotted over the cell-free secondary structure model.
