# Supplementary material for: Stress-induced changes in endogenous TP53 mRNA 5′ regulatory region
Source: J Biol Chem. 2025 Mar 18;301(4):108418. doi: 10.1016/j.jbc.2025.108418 (PMC12018109; doi:10.1016/j.jbc.2025.108418)
Supplement: Figure S4 [file mmc4.pdf]

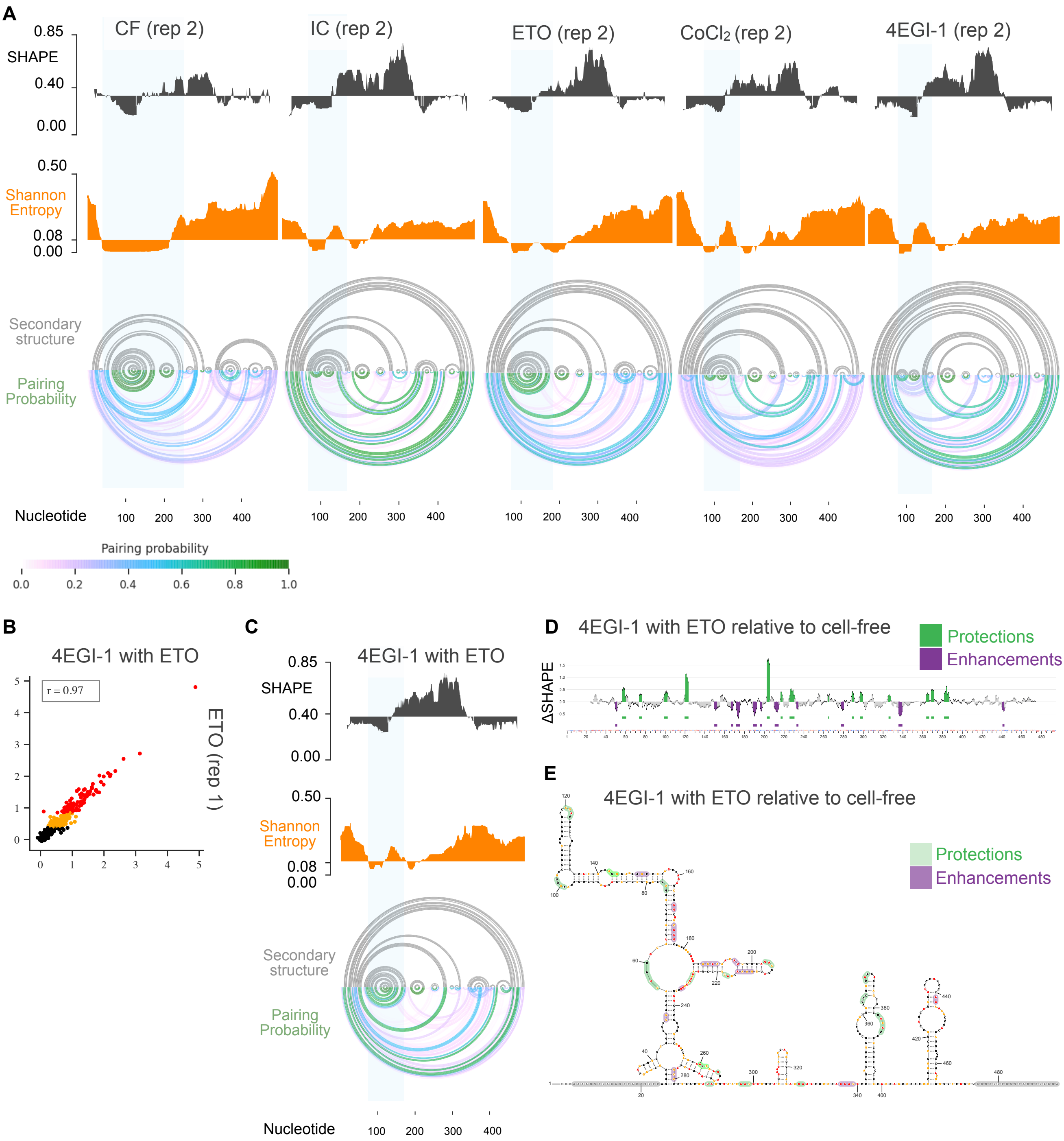

Figure S4. Replicate lowSS analysis and analysis of 4EGI-1 with etoposide. (A) LowSS analysis on replicate 2 for all conditions. (B) Pearson correlation of sample treated with 4EGI-1 and etoposide with sample treat with only etoposide. R value obtained is 0.97. (C) LowSS analysis for sample treated with 4EGI-1 and etoposide. (D) ΔSHAPE profile relative to cell-free for cells treated with a combination of 4EGI-1 and etoposide. (E) ΔSHAPE data from Figure S4D plotted on the cell-free secondary structure.
