# Supplementary material for: Stress-induced changes in endogenous TP53 mRNA 5′ regulatory region
Source: J Biol Chem. 2025 Mar 18;301(4):108418. doi: 10.1016/j.jbc.2025.108418 (PMC12018109; doi:10.1016/j.jbc.2025.108418)
Supplement: Figure S5 [file mmc5.pdf]

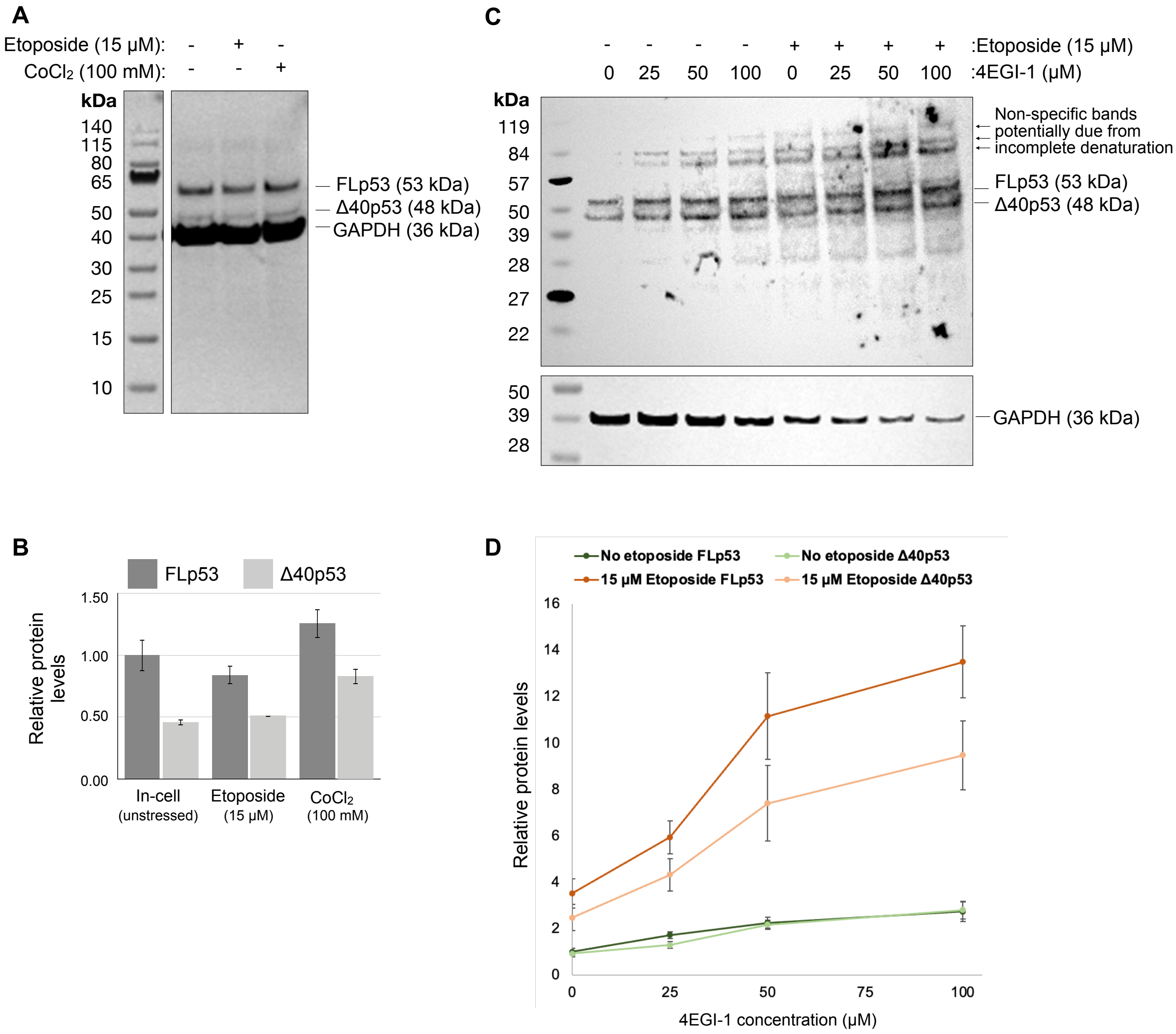

Figure S5. Western blotting using anti-p53 antibody measure full-length FLp53 and N-terminal truncated Δ40p53, along with anti-GAPDH antibody for GAPDH control for multiplexed detection. (A) Lane 1 was loaded with total protein from unstressed A549 cells, while lanes 2 and 3 were treated with 15 μM etoposide and 100 mM $CoCl_2$, respectively. (B) Relative protein levels from Figure S5A were quantified using densitometry with ImageJ conducted across three biological replicates. Ratios are normalized to GAPDH and relative to FLp53 in-cell unstressed. Standard deviations were calculated using three replicates. Changes in FLp53 and Δ40p53 across treatments were not statistically significant based on single factor ANOVA. (C) p53 protein levels for FLp53 and Δ40p53 are measured by western blotting over a gradient (0-100 μM) of cap-inhibitor (4EGI-1) treatment, in the absence and presence of 15 μM etoposide. After probing with anti-p53 the nitrocellulose membrane was stripped and re-probed with anti-GAPDH. (D) Relative protein levels of FLp53 and Δ40p53 from western blot of 4EGI-1 only and 4EGI-1 with etoposide treated cells were quantified using densitometry. Ratios are calculated normalized internally with GAPDH and relative to untreated control. Non-specific bands were not factored included in quantitation. Two technical replicates were used to calculate standard deviation and conduct one-way ANOVA based p-values. For no etoposide, p-values are 0.011 for FLp53 and 0.005 for Δ40p53, With etoposide, p-values are 0.0019 for FLp53 and 0.02 for Δ40p53.
