# Supplementary material for: Stress-induced changes in endogenous TP53 mRNA 5′ regulatory region
Source: J Biol Chem. 2025 Mar 18;301(4):108418. doi: 10.1016/j.jbc.2025.108418 (PMC12018109; doi:10.1016/j.jbc.2025.108418)
Supplement: Figure S6 [file mmc6.pdf]

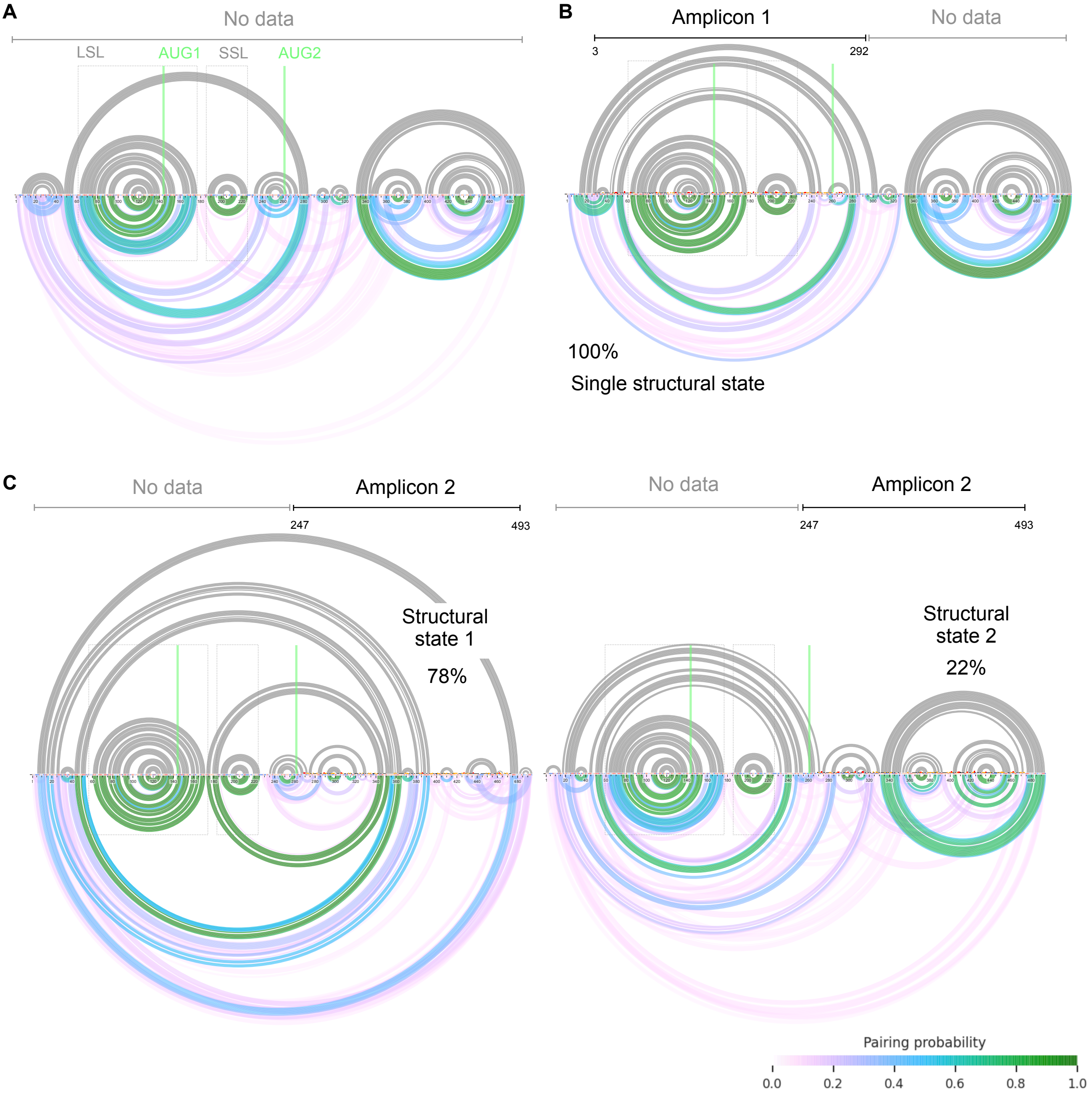

Figure S6. Dance-MaP deconvolution under in-cell unstressed condition. A. Structure prediction in the absence of any data through out the target length. LSL and SSL regions as well as locations of AUG1 and AUG2 are annotated for reference. B. In-cell DMS-MaP data collected for amplicon 1 (nucleotides 3-292) was used as input for dancemapper deconvolution algorithm. Only one structural state was detected within this amplicon. C. In-cell DMS-MaP data for amplicon 2 (247-493) was used as input for DanceMapper deconvolution producing two clusters representing structural states 1 and 2 at 78% and 22% distribution respectively.
