# Supplementary material for: Stress-induced changes in endogenous TP53 mRNA 5′ regulatory region
Source: J Biol Chem. 2025 Mar 18;301(4):108418. doi: 10.1016/j.jbc.2025.108418 (PMC12018109; doi:10.1016/j.jbc.2025.108418)
Supplement: Table S1 [file mmc7.pdf]

Table S1.

Between replicates

	%	Full target	lowSS region (60-250)
Cell-Free	sens	92.31	100
	ppv	91.67	100
In-cell	sens	100	100
	ppv	99.29	98.15
$CoCl_2$	sens	31.36	79.59
	ppv	30.94	86.67
Etoposide	sens	100	100
	ppv	99.29	98.15
4EGI-1	sens	97.64	92.00
	ppv	96.12	90.20

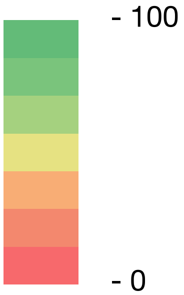

Relative to Cell-Free (Replicate 1) Structure Model

	%	Full target	lowSS region (60-250)
In-cell	sens	55.24	100
	ppv	56.43	100
$CoCl_2$	sens	25.87	42.59
	ppv	26.81	46.94
Etoposide	sens	54.55	98.15
	ppv	55.71	100
4EGI-1	sens	21.68	42.59
	ppv	24.41	46.00
4EGI-1 with etoposide	sens	54.55	98.15
	ppv	55.71	100

Table S1. Similarity scores of modeled secondary structures. Sensitivity (sens) and positive predictive value (ppv) calculated between replicates, and for all in-cell conditions relative to cell-free replicate 1 are listed. Calculations for full target and lowSS region (nucleotides 60-225) are provided.
