# Supplementary material for: Stress-induced changes in endogenous TP53 mRNA 5′ regulatory region
Source: J Biol Chem. 2025 Mar 18;301(4):108418. doi: 10.1016/j.jbc.2025.108418 (PMC12018109; doi:10.1016/j.jbc.2025.108418)
Supplement: Table S2 [file mmc8.pdf]

Table S2.

Amplicon	Forward primer	Start	Stop	Reverse primer	Start	Stop
1	AAAAGTCTAGAGCCACCGTCC	3	23	CAATATCGTCCGGGGACAGC	273	292
2	CCGTCCCAAGCAATGGATGA	247	266	**TCCCAGAATGCAAGAAGCCC**	474	493

Reverse transcription primer shown above in **bold**.

Illumina Adapter sequences:

GACTGGAGTTCAGACGTGTGCTCTTCCGATCTNNNNNNFORWARD-PRIMER
CCCTACACGACGCTCTTCCGATCTNNNNNNREVERSE-PRIMER

Table S2. Primers for gene-specific library preparation of *TP53* mRNA 5' end. Sequencing libraries were amplified for the target region 25-475 using two overlapping PCR products in a two-step process. In step 1, primers included adapter sequences. Step 2 primers added Illumina TruSeq DNA indexes. Reverse primer for amplicon 2 served as the reverse transcription primer for all samples.
