# Supplementary material for: Stress-induced changes in endogenous TP53 mRNA 5′ regulatory region
Source: J Biol Chem. 2025 Mar 18;301(4):108418. doi: 10.1016/j.jbc.2025.108418 (PMC12018109; doi:10.1016/j.jbc.2025.108418)
Supplement: Table S3 [file mmc9.pdf]

Table S3.

Sample	Median depth		95th percentile mutation rate (%)	
	Modified (5NIA)	Untreated (DMSO)	Modified (5NIA)	Untreated (DMSO)
Cell free				
Replicate 1	140313	111967	3.61	0.68
Replicate 2	93247	131167	3.67	0.89
In-cell				
Replicate 1	134150	132128	3.28	1.56
Replicate 2	115331	101886	2.4	0.9
Etoposide				
Replicate 1	90757	110876	2.61	0.83
Replicate 2	153613	131697	2.51	0.8
$CoCl_2$				
Replicate 1	83677	60188	3.02	0.96
Replicate 2	147956	83575	3.18	0.76
4EGI-1				
Replicate 1	124919	118942	2.89	0.89
Replicate 2	143626	124000	2.98	1.07
4EGI-1-ETO	141414	127231	2.05	1.04

Table S3. Read depth and mutation rates for all conditions. Median read depths and 95th percentile mutation rates for each condition including replicates are listed. Per-nucleotide read-depth and mutation rate are included in supplementary materials.
